# Transcriptional Regulation of PIK3CD and PIKFYVE in T-Cell Acute Lymphoblastic Leukemia by IKAROS and Protein Kinase CK2

**DOI:** 10.3390/ijms22020819

**Published:** 2021-01-15

**Authors:** Elanora Dovat, Chunhua Song, Tommy Hu, Mohammad Atiqur Rahman, Pavan Kumar Dhanyamraju, Morgann Klink, Daniel Bogush, Mario Soliman, Shriya Kane, Mary McGrath, Yali Ding, Dhimant Desai, Arati Sharma, Chandrika Gowda

**Affiliations:** 1 Department of Pediatrics, Pennsylvania State University College of Medicine, Hershey, PA 17033, USA; edovat@pennstatehealth.psu.edu (E.D.); chunhua.song@osumc.edu (C.S.); tommyhu@pennstatehealth.psu.edu (T.H.); mrahman@pennstatehealth.psu.edu (M.A.R.); pdhamyamraju@pennstatehealth.psu.edu (P.K.D.); mreed7@pennstatehealth.psu.edu (M.K.); dbogush@pennstatehealth.psu.edu (D.B.); soliman.mario1@gmail.com (M.S.); shriya.kane96@gmail.com (S.K.); marymcgra17@gmail.com (M.M.); yding@pennstatehealth.psu.edu (Y.D.); 2 Ohio State University School of Medicine, Columbus, OH 43210, USA; 3 Department of Pharmacology, Pennsylvania State University College of Medicine, Hershey, PA 17033, USA; ddesai@pennstatehealth.psu.edu (D.D.); asharma@pennstatehealth.psu.edu (A.S.)

**Keywords:** IKAROS, protein kinase CK2, T cell acute lymphoblastic leukemia, PI3K, transcriptional regulation

## Abstract

IKAROS, encoded by the *IKZF1* gene, is a DNA-binding protein that functions as a tumor suppressor in T cell acute lymphoblastic leukemia (T-ALL). Recent studies have identified IKAROS’s novel function in the epigenetic regulation of gene expression in T-ALL and uncovered many genes that are likely to be directly regulated by IKAROS. Here, we report the transcriptional regulation of two genes, phosphatidylinositol-4,5-bisphosphate 3-kinase catalytic subunit delta (*PIK3CD*) and phosphoinositide kinase, FYVE-type zinc finger containing (*PIKFYVE*), by IKAROS in T-ALL. *PIK3CD* encodes the protein p110δ subunit of phosphoinositide 3-kinase (PI3K). The PI3K/AKT pathway is frequently dysregulated in cancers, including T-ALL. IKAROS binds to the promoter regions of PIK3CD and PIKFYVE and reduces their transcription in primary T-ALL. Functional analysis demonstrates that IKAROS functions as a transcriptional repressor of both PIK3CD and PIKFYVE. Protein kinase CK2 (CK2) is a pro-oncogenic kinase that is overexpressed in T-ALL. CK2 phosphorylates IKAROS, impairs IKAROS’s DNA-binding ability, and functions as a repressor of PIK3CD and PIKFYVE. CK2 inhibition results in increased IKAROS binding to the promoters of PIK3CD and PIKFYVE and the transcriptional repression of both these genes. Overall, the presented data demonstrate for the first time that in T-ALL, CK2 hyperactivity contributes to PI3K signaling pathway upregulation, at least in part, through impaired IKAROS transcriptional regulation of PIK3CD and PIKFYVE. Targeting CK2 restores IKAROS’s regulatory effects on the PI3K oncogenic signaling pathway.

## 1. Introduction

IKAROS is a zinc finger protein encoded by the *IKZF1* gene. IKAROS binds to DNA and functions as a transcriptional regulator of its target genes via chromatin remodeling [1]. IKAROS-knockout mice develop T cell malignancy with 100% penetrance [2]. Inactivation of IKAROS by a recurrent genetic alteration in the *IKZF1* gene is seen in nearly 4–5% of adult and pediatric T- cell Acute Lymphoblastic Leukemia (T-ALL) and is associated with poor outcome [3,4,5,6]. Early T cell precursor (ETP) leukemia is a distinct subtype of T-ALL, with a worse outcome, in which nearly 11% of cases show *IKZF1* alterations. IKAROS plays a central role in hematopoiesis, lymphoid development, and T cell differentiation [7,8]. Recently published studies have established IKAROS as a global epigenetic regulator of gene expression in T-ALL [9,10]. Global epigenomic analyses in T-ALL have shown that IKAROS functions as a tumor suppressor by widespread sequence-specific DNA binding to regulatory elements of its target genes and recruitment of histone-remodeling complexes, thereby repressing or activating gene transcription [10,11]. We used published genomic data to identify possible IKAROS target genes. IKAROS-mediated transcriptional regulation of oncogenic signaling pathways in T-ALL is not entirely understood. Here we present the identification and validation of several genes of the phosphoinositide 3-kinase (PI3K) pathway that are likely to be directly regulated by IKAROS.

**Figure 1:**
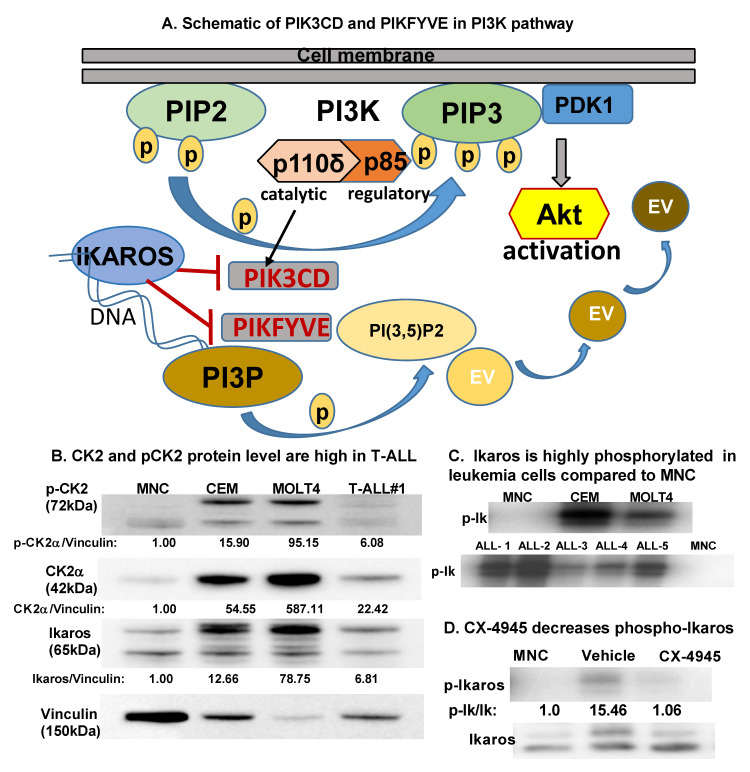
**CK2 is overexpressed in T-ALL compared to normal mononuclear cells**. (**A**) Schematic showing PIK3CD and PIKFYVE genes in the PI3K pathway. (**B**) Baseline protein levels of CK2α, pCK2, and IKAROS in the T cell leukemia cell panel (CCRF-CEM, MOLT4, and primary T-ALL cells (labeled T-ALL#1)) were measured by Western blot and compared to peripheral blood mononuclear cells (MNCs). The protein level is graphed relative to vinculin as a loading control. (**C**) Radio-immunoblot showing phospho-IKAROS in a leukemia cell panel (CCRF-CEM, MOLT4, and primary T-ALL cells (labeled ALL#1-5)) compared to MNCs. (**D**) Radio-immunoblot showing a decrease in the phospho-IKAROS level following CX-4945 treatment. MOLT4 cells were treated with 10 μM of CX-4945 for 24 h. EV, endosomal vesicle; PIK3CD, phosphatidylinositol 4,5-bisphosphate 3-kinase catalytic subunit delta; and PIKFYVE, phosphoinositide kinase, FYVE-type zinc finger containing; PI3K, phosphoinositide 3-kinase; P, phospho; PIP2, phosphatidylinositol 4,5-bisphosphate; PIP3, phosphatidylinositol 3,4,5-trisphosphate; PDK1, phosphoinositide-dependent kinase; PI3P, phosphatidylinositol-3-phosphate; PI(3,5)P2, phosphotydile inositol 3,5-biphosphate.

In cancer, including T-ALL, the PI3K pathway is often dysregulated [12,13,14,15]. Here, we report IKAROS-mediated transcriptional regulation of two PI3K pathway genes, phosphatidylinositol 4,5-bisphosphate 3-kinase catalytic subunit delta (*PIK3CD*) and phosphoinositide kinase, FYVE-type zinc finger containing (*PIKFYVE*). PIK3CD encodes the protein p110δ isoform of the catalytic subunit of PI3K [16]. Class IA PI3Ks are heterodimers consisting of a catalytic subunit (p110) and a regulatory subunit (p85). Three different genes can encode each subunit. Following activation by growth factors or ligand binding to receptor tyrosine kinases, PI3Ks phosphorylate phosphatidylinositol 4,5-bisphosphate (PIP2) to generate phosphatidylinositol 3,4,5-trisphosphate (PIP3) [17]. PIP3 plays a key role by recruiting pleckstrin homology (PH) domain-containing proteins such as AKT (also known as Protein Kinase B) and phosphoinositide-dependent kinase 1 (PDK1) to the plasma membrane. These events activate the signaling cascade to promote cell growth, survival, and proliferation [18]. Class I PI3K plays a central role in mediating receptor tyrosine kinase-induced AKT signaling and is often activated in cancers, including T-ALL [18]. Targeting the PI3K-AKT pathway in hematological malignancies shows promising results [19]. PIKFYVE encodes an enzyme that phosphorylates the D-5 position in phosphotidylinositol and phosphatidylinositol-3-phosphate (PI3P) to form PtdIns5P and PtdIns 3,5 biphosphate (PI(3,5)P2). PIKFYVE lipid kinase plays an important role in regulating endomembrane homeostasis and forming endosome carrier vesicles from early endosomes. PIKFYVE-mediated regulation of endosomal PI3P and PI(3,5)P2 levels controls Toll-like receptor (TLR) signaling [20]. Inhibition of PIKFYVE kinase using small molecules shows efficacy in treating autoimmune diseases, inflammatory disorders, viral infections and cancer [21] (Figure 1A).

Protein kinase CK2 (CK2) is a pro-oncogenic kinase overexpressed in many cancers, including T-ALL [22,23]. CK2 is a ubiquitous and constitutively active serine–threonine kinase [24]. CK2 exists as a tetramer with two catalytic subunits (CK2α and CK2α’) and two regulatory subunits (β). CK2 is essential for vital cellular processes and embryonic development [25,26]. CK2 overexpression in B cell and T cell lineage acute lymphoblastic leukemia (ALL), chronic lymphocytic leukemia (CLL), chronic myelogenous leukemia (CML), myeloproliferative neoplasm (MPN), and acute myeloid leukemia (AML) promotes cell survival and imparts resistance to apoptosis via several mechanisms [23,27]. Targeting CK2 using pharmacological inhibitors selectively renders tumor cells highly dependent on its activity susceptible to cell death [28]. A potent and selective, ATP-competitive, small-molecule CK2 inhibitor known as 5-(3-chlorophenyl) amino) benzo [c] naphthyridine-8-carboxylic acid (CX-4945) has shown favorable tolerability and a toxicity profile in cancer patients [29,30,31]. CX-4945 (silmitasertib) is currently in a phase I/II clinical trial for patients with recurrent medulloblastoma (NCT03904862) and advanced basal cell carcinoma (NCT03897036). CK2-driven post-translational modification of transcription factors and tumor suppressors, such as phosphatase and tensin homolog (PTEN), P53, IKAROS, and promyelocytic leukemia protein (PML) [19,32,33], often results in impaired transcriptional activity. This leads to the overactivation of oncogenic signaling pathways that are typically kept in check by tumor suppressors. CK2 inhibitors, including CX-4945, show efficacy in preclinical models of T-ALL [32].

CK2-mediated phosphorylation of IKAROS impairs its DNA binding and disrupts IKAROS’s function as a repressor or activator of transcription [34,35]. In B-cell ALL, inhibition of CK2 restores IKAROS’s DNA binding and transcriptional regulation of target genes [36,37]. However, the role of IKAROS in transcriptional regulation of signaling networks in T-ALL is not entirely understood. A recent study revealed that IKAROS functions as a global epigenomic regulator in T-ALL. Here, we report that in T-ALL with high CK2 expression, IKAROS is significantly phosphorylated. We show that IKAROS regulates essential genes in the PI3K pathway. Inhibition of CK2 in T-ALL restores the DNA-binding ability of the IKAROS tumor suppressor and restores IKAROS’s ability to repress PI3K pathway genes.

## 2. Results

### 2.1. CK2 Expression Increases in T-ALL and Correlates with an Elevated Level of Phosphorylated IKAROS

We examined protein levels of CK2α and phospho-CK2α in a panel of T-ALL cells using Western blot. As shown in previous studies [22,32,38], we found increased expression of the CK2α protein in T cell leukemia cells compared to normal peripheral blood mononuclear cells (MNCs) (Figure 1B). Phosphorylated IKAROS (p-IKAROS) was measured in CEM, MOLT4, and primary T-ALL cells using radio-immunoblot. CK2α expression correlated with increased phosphorylated IKAROS in T-ALL cells (Figure 1C). We used MOLT4, CEM, and T-ALL#1 cells for our study based on increased CK2α expression.

### 2.2. CK2 Inhibitor CX-4945 Decreases Phosphorylated IKAROS in T-ALL

In B cell ALL, phosphorylation of IKAROS by CK2 impairs IKAROS DNA binding, pericentromeric localization, ubiquitination, and IKAROS protein degradation. Targeting CK2 using pharmacological inhibitors decreases IKAROS’s phosphorylation and restores the DNA-binding ability of IKAROS [36]. We subjected T-ALL cells with high baseline p-IKAROS to the CK2 inhibitor CX-4945 at IC50 (inhibitory concentration) for 48 h. Radiolabeling of cells using P32 followed by IKAROS immunoprecipitation and radio-immunoblot showed that CX-4945 treatment decreases phosphorylated IKAROS in MOLT4 cells (Figure 1D).

### 2.3. IKAROS Binds to the Promoters of PIK3CD and PIKFYVE in T-ALL Cells

**Figure 2:**
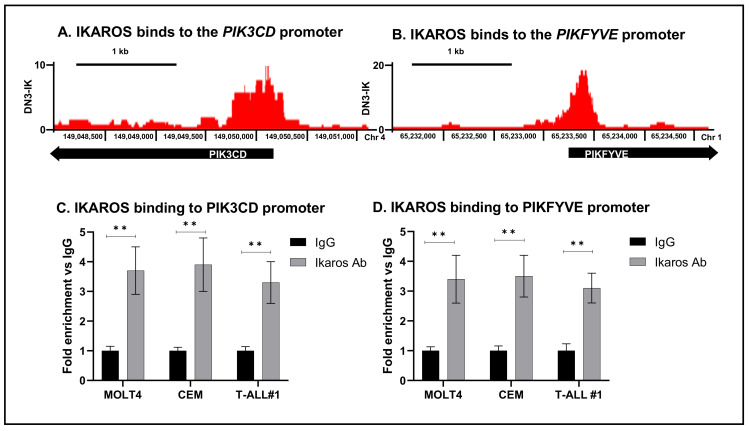
**IKAROS binds to the promoter regions of PIK3CD and PIKFYVE**. Chromatin immunoprecipitation (ChIP) followed by next-generation sequencing (ChIP-seq) and analysis of genome-wide occupancy of IKAROS was performed on DN3 cells (IKAROS-null T-ALL cells) following IKAROS re-introduction. IKAROS global DNA binding was analyzed on days 1, 2, and 3 following IKAROS introduction. (**A**) ChIP-seq signal map for IKAROS binding to the *PIK3CD* promoter region on day 1. (**B**) ChIP-seq signal map for IKAROS binding to the *PIKFYVE* promoter region on day 1. The *y*-axis represents a log 2 fold change enrichment of IKAROS binding (** *p* < 0.01). CEM, MOLT4, and T-ALL#1 cells were treated with 10 μM of CX-4945 for 24 h. IKAROS binding to the *PIK3CD* (**C**) and *PIKFYVE* (**D**) promoter region was confirmed using qChIP assay in vehicle- and CX-4945-treated cells. Results are mean +/– SD of triplicates representative of one of three independent experiments.

IKAROS is a critical tumor suppressor in T-ALL [39]. Deleting one copy of IKAROS (haplo-knockout mice) gives rise to T-ALL that is 100% transmitted to the next generation of mice [2]. Recently, Ding et al. described global epigenomic regulation by IKAROS in DN3 cells following IKAROS reintroduction. Using chromatin immunoprecipitation followed by next-generation sequencing (ChIP-seq), the authors identified firm binding peaks of IKAROS in the promoter region of several target genes. Introduction of IKAROS into IKAROS-null T-ALL cells (double-negative CD4-, CD8-, and DN3 cells) results in T cell differentiation and decreased proliferation. We analyzed publicly available ChIP-seq data (Gene Expression Omnibus database with accession no. GSE126391) showing global IKAROS DNA occupancy in DN3 (IKAROS-null T-ALL) following IKAROS introduction. The results showed increased IKAROS-binding peaks at regulatory elements of *PIK3CD* and *PIKFYVE*, defined as transcription start site ± 3 kilobases (Figure 2A,B). We identified 5642 prospective genes regulated by IKAROS, including PIKFYVE and PIK3CD. The IKAROS consensus-binding site was noted as GGAA and GGGA. *PIK3CD* and PIKFYVE were selected for further analysis as they are important members of the PI3K pathway and showed a significant increase in IKAROS DNA occupancy, suggesting that IKAROS may regulate the transcription of these genes. The PIK3CD gene encodes the delta isoform of the catalytic subunit p110 of the PI3K enzyme (Figure 1D). The PIKFYVE gene encodes a protein that functions as a lipid kinase essential for endosome vesicle formation and intracellular signal transmission (Figure 1D). IKAROS binding to the promoter of PIK3CD and PIKFYVE was further confirmed by quantitative chromatin immunoprecipitation (qChIP) in human T-ALL cell lines MOLT4 and CEM and primary T-ALL cells labeled T-ALL#1 (Figure 2C,D). Human embryonic kidney (HEK) 293T cells were used as a negative control since they do not contain IKAROS and do not show increased DNA binding.

### 2.4. IKAROS Negatively Regulates Transcription of PIK3CD and PIKFYVE Genes

**Figure 3:**
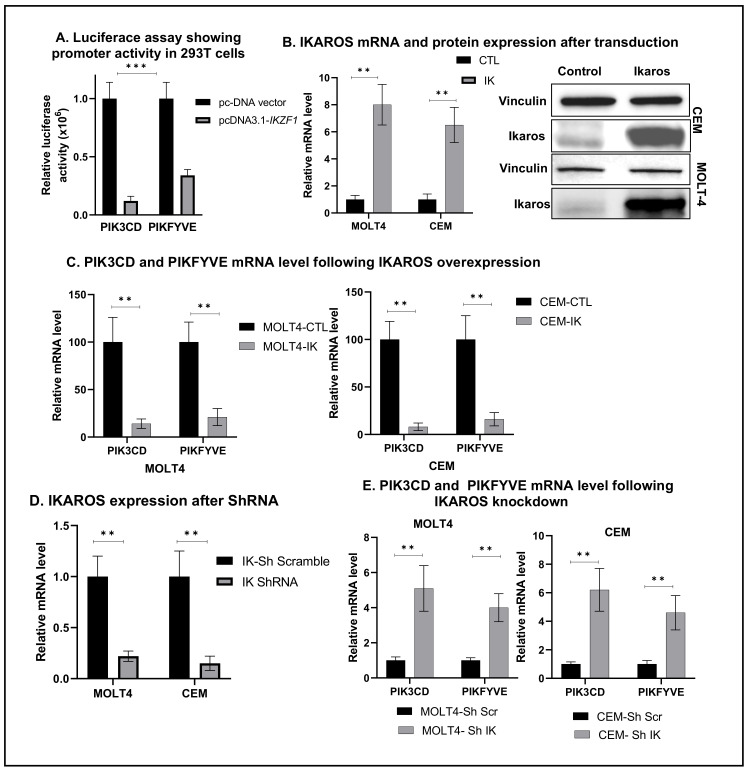
**IKAROS represses PIK3CD and PIKFYVE gene transcription in T-ALL**. Luciferase reporter assay was performed on HEK 293T cells transfected with the IKZF1 plasmid (pcDNA 3.1-IK) or a control vector (pcDNA3.1). The result in (**A**) shows repression of the PIK3CD and PIKFYVE luciferase promoter construct by the IKAROS-expressing vector pcDNA3.1-IK in comparison to the pcDNA3.1 empty vector control in HEK 293T cells. (**B**) CEM and MOLT4 cells were transduced to express IKZF1 (MIG-IK) or with an empty vector (MIG-CTL). Relative mRNA expression of IKAROS following transduction. (**C**) mRNA level of PIK3CD and PIKFYVE in IKAROS-overexpressed (left panel) MOLT4 and (right panel) CEM cells assessed using qRT-PCR. (**D**) MOLT4 and CEM cells were treated with IKZF1 shRNA (shIK) or scrambled shRNA control (shCTL). The relative expression of IKZF1 (left panel) assessed by qRT-PCR. (**E**) mRNA level of PIK3CD and PIKFYVE in MOLT4 (left panel) and CEM (right panel) cells. Results are mean ± SD of triplicates representative of one of three independent experiments. The *p*-value summaries are as follows: *p* < 0.01 (**); *p* < 0.001 (***).

We used a luciferase reporter assay to determine whether IKAROS binding to the *PIK3CD* and *PIKFYVE* promoter region alters gene expression. We performed transient co-transfection of the PIK3CD or PIKFYVE promoter region fused with the reporter gene and *IKZF1* in HEK 293T cells. The results showed that IKAROS represses the promoter activity of PIK3CD and PIKFYVE compared to the negative control (Figure 3A). These results demonstrated that IKAROS can repress transcription by directly binding to the promoters of *PIK3CD* and *PIKFYVE* genes.

Further, we performed functional analysis using *IKAROS*-overexpressed and *IKAROS*-silenced T-ALL cells to determine the functional importance of IKAROS binding to DNA at the promoters of *PIK3CD* and *PIKFYVE*. IKAROS was overexpressed in MOLT4 and CEM cells by transduction of a retrovirus expressing wild-type *IKZF1* and an empty vector as a control (Figure 3B). Overexpression of IKAROS in MOLT4 and CEM cells decreased the messenger RNA levels of PIK3CD and PIKFYVE (Figure 3C). IKAROS was silenced by treating MOLT4 and CEM cells with *IKZF1* shRNA (Figure 3D). We used T-ALL cells treated with scrambled shRNA (short hairpin control- shCTL) as a control. IKAROS knockdown resulted in increased mRNA levels of *PIK3CD* and *PIKFYVE* (Figure 3E). These results establish the role of IKAROS as a transcriptional repressor of *PIK3CD* and *PIKFYVE*.

### 2.5. Inhibition of CK2 Restores IKAROS DNA-Binding and Transcriptional Repression of PIK3CD and PIKFYVE

**Figure 4:**
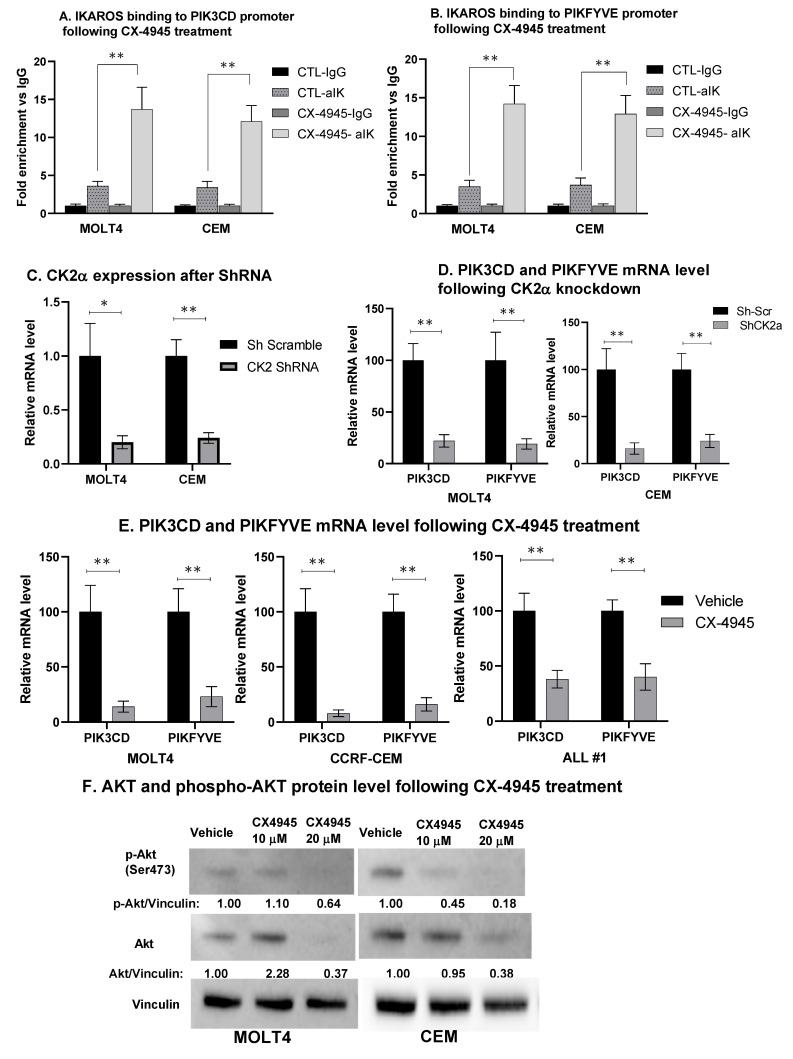
**CK2 inhibition restores IKAROS DNA binding and repression of PIK3CD and PIKFYVE**. MOLT4, CEM, and T-ALL#1 cells were treated with 10 μM of CX-4945 for 24 h. IKAROS binding to the (**A**) PIK3CD and (**B**) PIKFYVE promoter region was confirmed using qChIP assay in vehicle- and CX-4945-treated cells. Results are mean ± SD of triplicates representative of one of three independent experiments. Molecular inhibition of CK2α in CEM and MOLT4 cells was achieved using shRNA. Two of four shRNAs showed a significant and similar decrease in CK2α. qRT-PCR shows the mRNA level of (**C**) CK2α and (**D**) PIK3CD and PIKFYVE in CK2-silenced MOLT4 (left panel) and CEM (right panel) cells. CEM and MOLT4 cells were treated with 10 and 20 μM of CX-4945 for 48 h, and T-ALL#1 primary leukemia cells were treated with 10 μM of CX-4945 for 12 h. mRNA and protein were extracted. (**E**) The mRNA level of PIK3CD and PIKFYVE was measured in CX-4945-treated CEM, MOLT4, and T-ALL#1 cells. (**F**) AKT and phosphorylated-AKT (p-AKT) protein levels were measured by Western blot. The protein level is expressed relative to vinculin. The *p*-value summaries are as follows: *p* ≤ 0.05 (*); *p* < 0.01 (**).

Phosphorylation severely hampers IKAROS’s ability to bind DNA and regulate the transcription of genes [35]. CK2 phosphorylates IKAROS at several serine–threonine sites. In T-ALL, overexpression of CK2 correlates with increased phosphorylation of IKAROS. Treatment with CX-4945 restored IKAROS binding to the promoters of *PIK3CD* and *PIKFYVE* genes, as shown in the qChIP assay (Figure 4A,B). We achieved molecular inhibition of CK2α by knockdown with short hairpin RNA (shRNA) directed against the CK2α catalytic subunit (CSNK2A1) (Figure 4C). CK2 inhibition decreased the mRNA expression of PIK3CD and PIKFYVE (Figure 4D). Treatment of MOLT4 and CEM cells with CX-4945 also decreased the mRNA level of PIK3CD and PIKFYVE (Figure 4E) and the protein level of downstream targets of PI3K-AKT (Figure 4F). These results suggested that inhibition of CK2 restores IKAROS binding to DNA and IKAROS-mediated repression of *PIK3CD* and *PIKFYVE* genes.

### 2.6. IKAROS Regulates PIK3CD and PIKFYVE Gene Expression via Chromatin Remodeling

**Figure 5:**
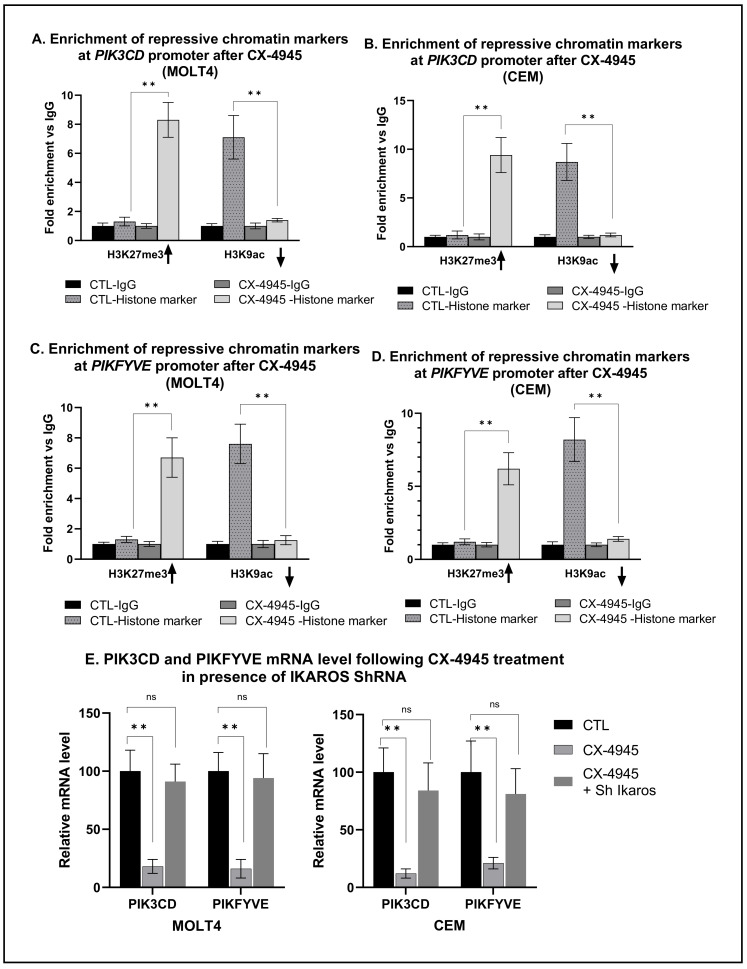
**IKAROS regulates the expression of PIK3CD and PIKFYVE by repressive chromatin formation**. The qChIP assay showed enrichment of histone markers at PIK3CD (**A**,**C**) and PIKFYVE (**B**,**D**) promoters. The qChIP assay was performed using MOLT4 (**A**,**B**) and CEM (**C**,**D**) cells (treated with 10 μM of CX-4945 for 48 h) to determine the fold enrichment of histone markers H3K27me3 and H3K4me3 at PIK3Cd and PIKFYVE promoters compared to control cells. (**E**) MOLT4 and CEM cells were treated with IKZF1 shRNA (shIK) or scrambled shRNA control (shCTL). IKAROS-knockdown CEM and MOLT4 cells were then treated with 5 μM of CX-4945 for 48 h. Changes in PIK3CD and PIKFYVE gene expression were measured using qPCR. The *p*-value summaries are as follows: *p* < 0.01 (**).

Transcriptional regulation of target genes by IKAROS often involves chromatin remodeling [11]. Chemical modifications, such as methylation and acetylation of the histone proteins present in chromatin, influence gene expression by changing the accessibility of chromatin to transcription. A specific modification (acetylation or methylation) of a specific histone protein is called a histone mark. The H3K9Ac histone mark is the acetylation of lysine 9 (K9) of the H3 histone protein. The H3K27me3 histone mark is the methylation of lysine 27 (K27) of the H3 histone. H3K9 acetylation (H3K9Ac) is a marker of open and active chromatin. H3K27 tri-methylation (H3K27me3) is a marker of closed and repressive chromatin. Enrichment of H3K27me3 and loss of H3K9Ac represent a repressive chromatin signature [40]. The mechanism through which IKAROS represses the transcription of PIK3CD and PIKFYVE is likely chromatin remodeling. To determine that, we performed serial qChIP assays to determine chromatin signature changes following CX-4945 treatment of T-ALL cells. CX-4945 treatment of MOLT4 and CEM cells resulted in enrichment of H3K27me3 (Figure 5A,C) and loss of H3K9ac (Figure 5B,D) histone modification markers at the promoters of PIK3CD and PIKFYVE genes compared to the negative control. The results suggest that IKAROS represses the transcription of PIK3CD and PIKFYVE genes by inducing the formation of repressive chromatin at the promoters of these genes.

### 2.7. CX-4945-Induced Repression of PI3K Pathway Genes Is Mediated via IKAROS

CK2 is a promiscuous kinase with many substrates, IKAROS being one of them [41]. To test whether IKAROS is essential for CK2 inhibitor-mediated repression of genes, we treated MOLT4 and CEM cells containing IKAROS shRNA with CX-4945. The mRNA level of PIK3CD and PIKFYVE was measured using qPCR. The results showed that CX-4945 treatment failed to decrease the mRNA level of PIK3CD and PIKFYVE in IKAROS shRNA-treated cells (Figure 5E). These results showed that the CK2-mediated regulation of PIK3CD and PIKFYVE gene expression is IKAROS-dependent.

## 3. Discussion

Several studies have established IKAROS tumor suppressor activity in T-ALL [7,8,42]. However, a detailed evaluation of IKAROS target genes and signaling networks regulated by IKAROS and CK2 in T-ALL is lacking. Meta-analyses of published ChIP-seq data of the IKAROS-null T-ALL cell line (DN3) following IKAROS reintroduction showed that IKAROS binds to many gene regulatory elements, potentially regulating their expression.

**Figure 6:**
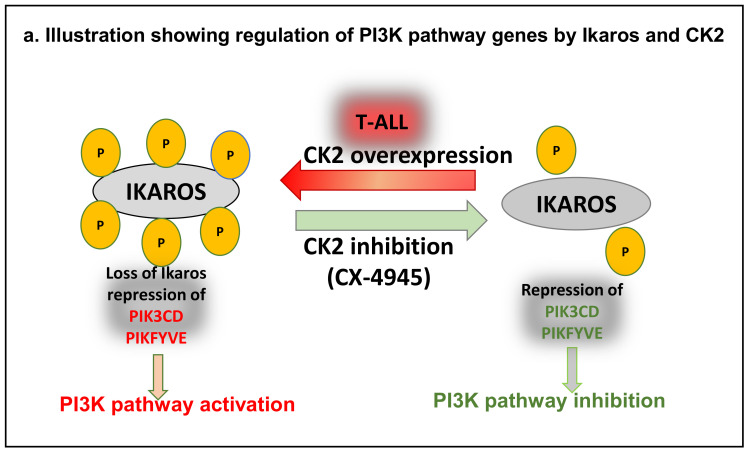
Model illustration of regulation of PI3K pathway genes PIK3CD and PIKFYVE in T-ALL by CK2 and IKAROS.

We found that phosphorylated IKAROS is higher in T cell leukemia cells compared to normal mononuclear cells. CK2 protein expression increased in most T cell leukemia samples tested, and increased CK2 correlated with phosphorylated IKAROS. In addition to genetic inactivation, post-translational modification of IKAROS by CK2-mediated phosphorylation can lead to IKAROS’s functional inactivation [35,43]. The IKAROS transcription factor’s regulatory functions depend on its ability to localize to pericentromeric heterochromatin and bind to DNA [44,45]. These processes are impaired following phosphorylation by CK2 [36]. In T-ALL, we showed that increased CK2 levels correlate with high levels of phosphorylated IKAROS. Using T-ALL cell lines and primary T-ALL cells, we showed that IKAROS binds to the regulatory elements of two genes from the PI3K pathway, *PIK3CD* and *PIKFYVE*, and represses their expression via direct binding as well as the formation of a repressive chromatin signature at the promoter region (Figure 6).

Inhibition of CK2 restored IKAROS binding to DNA at the promoters of *PIK3CD* and *PIKFYVE* and further resulted in the repression of *PIK3CD* and *PIKFYVE* expression. Our results revealed a novel mechanism of regulation of PI3K pathway genes by CK2 and IKAROS (Figure 6). Further studies are required to uncover other signaling networks and oncogenic signaling pathways regulated by IKAROS and CK2 in T-ALL. A clear understanding of the crosstalk between IKAROS and CK2 will aid in developing more effective combination therapies for the treatment of T-ALL.

The CK2 inhibitor CX-4945 has shown promising preclinical activity as a single agent and in combination with chemotherapy agents in T-ALL murine models [22,32]. The mechanism of action of CK2 inhibitors in T-ALL is not entirely understood. Data are lacking regarding the efficacy of CK2 inhibitors in patients with T-ALL, with and without *IKZF1* deletions. Therefore, the applicability/usefulness of CK2 inhibitors in T-ALL patients should be further investigated, as the degree of response could be dependent on *IKZF1* deletion and PI3K pathway alterations. These results reveal a potential novel mechanism of action of CX-4945 in T-ALL via restoration of IKAROS-mediated repression of PI3K genes PIK3CD and PIKFYVE.

## 4. Materials and Methods

### 4.1. Cells and Cell Culture

HEK 293T, CEM, and MOLT4 cells were obtained from the American Type Culture Collection (ATCC). De-dentified patient samples were provided by Loma Linda University (Loma Linda, CA), Penn State College of Medicine (Department of Pediatrics Developmental Therapeutics and Preclinical Core (DTPC)), and Penn State Cancer Institute collected under an approved material transfer agreement (MTA) and after approval from institutional review board (IRB). CEM, MOLT4, and primary T-ALL cells were cultured or maintained in RPMI 1640 medium (Corning) supplemented with 10% fetal bovine serum (Hyclone) and incubated at 37 °C in a humidified atmosphere of 5% carbon dioxide. HEK 293T cells were cultured in Dulbecco’s Modified Eagle Medium -DMEM (CellGro) supplemented with 10% fetal bovine serum (FBS).

### 4.2. Reagents

CK2 inhibitor CX-4945 sodium salt was purchased from MedChem Express (Monmouth Junction, NJ, USA).

### 4.3. Meta-Analysis

IKAROS binding at PIK3CD and PIKFYVE promoters in DN3 T-ALL cells following expression of IKAROS was determined by analyzing genome-wide IKAROS ChIP-seq data made previously available by Y. Ding and S. Dovat in the Gene Expression Omnibus (GEO) database (accession no. GSE126391).

### 4.4. In Vitro Phospho-IKAROS Labeling

CEM, MOLT4, and primary T-ALL cells were incubated with 0.5 mCi/mL [32P] of orthophosphate (PerkinElmer, Waltham, MA, USA) in phosphate-free RPMI 1640 medium for 6 h. MOLT4 cells were treated with 10 μM of CX-4945 for 48 h before incubating with orthophosphate. Nuclear protein was extracted, and IKAROS was immuno-precipitated using the Dynabead Protein G Immunoprecipitation Kit (Thermo Fisher Scientific) according to the manufacturer’s protocol. As described previously [35], IKAROS was eluted, separated by SDS-PAGE, transferred to a membrane, and imaged by radiography.

### 4.5. Quantitative Chromatin Immunoprecipitation (qChIP)

qChIP assays for IKAROS binding in T-ALL cells and qChIP assays for H3K4me3, H3K9me3, and H3K9ac histones were performed, as described previously [36]. The qChIP primers used to link immuno-precipitated DNA to the promoter were as follows:

PIK3CD: F5′-TCCCCGGCAATCATAGCA-3′; R5′-CTGGGTTTTTTATTTTTTCCATCT-3′

PIKFYVE: F5′-CCTAATCTCGGCCAAAAGATCA-3′; R5′-TCAGATGCTCAGGCAGAAGGA-3′

### 4.6. Quantitative RT-PCR

RNA isolation, cDNA generation, and qPCR were performed, as described previously [36]. The primers used in this study were as follows: 

18s RNA: GTAACCCGTTGAACCCCATT (sense)

CSNK2A1 (CK2): AGCGATGGGAACGCTTTG (sense) and AAGGCCTCAGGGCTGACAA (antisense)

IKZF1: GGCGCGGTGCTCCTCCT (sense) and TCCGACACGCCCTACGACA (antisense)

PIK3CD: TTCAGCTTCCCCGATTGC (sense) and CAGCTCATCGTCCGTCAGTTT (antisense)

PIKFYVE: CACAAGGGCACAAGCTATAGCA (sense) and ACAATCCAGCCAACGTCCAT (antisense)

### 4.7. Western Blot

CEM and MOLT4 cells were treated with 5 or 10 μM of CX-4945 or DMSO control for up to 48 h, T-ALL#1 cells were treated with CX-4945 for 24 h, and whole-cell lysate was collected. Protein was quantified using the Bradford assay and used for Western blot analysis and immunoblot. Western blots were performed using anti-CK2α (Cat# sc-373894) and AKT (Cat# sc-5298) from Santa Cruz Biotechnology (Dallas, TX, USA), p-CK2 (Cat# 8738) and p-A (Ser473) (CST Cat# 4058) from Cell Signaling Technology (Danvers, MA, USA), IKAROS (Cat# 66966-1-Ig) from Proteintech (Rosemont, IL, USA), or vinculin (Cat# 700062) from Thermo Fisher Scientific, as described previously [36].

### 4.8. IKZF1 (IKAROS) and CSNKI2A1 (CK2α) Knockdown

We used a neon transfection system and pGP-V-RS shRNA plasmids (Origene) for IKAROS (*IKZF1*) and CK2α (*CSNK2A1*) knockdown in CEM and MOLT4 cells, as described previously [36]. We confirmed IKAROS and CK2α using qRT-PCR and Western blot.

### 4.9. Retroviral Transduction

Transduction of CEM and MOLT4 cells with retroviruses produced by transient transfection using pMIG-CTL or pMIG-*IKZF1* was performed, as described previously [36]. Green fluorescent protein (GFP)+ cells were sorted using a FACS Aria SORP (Becton Dickinson) instrument. Sorted cells were further cultured using the above conditions. The construction of expression plasmids was as described previously [36].

### 4.10. Luciferase Reporter Assay

PIK3CD or PIKFYVE promoter-driven luciferase reporter (150 ng) activity was assessed in CEM and MOLT4 cells co-transfected with the expression plasmid for IKAROS (pcDNA3.1 IKAROS −150 ng) using Lipofectamine 2000 (Invitrogen). The pcDNA3.1 plasmid was co-transfected as a control of transfection efficiency. After 24 h transfection, luciferase assays were performed, as described previously [36]. Luciferase activities were calculated as a fold change relative to vector-only cells and normalized to pcDNA3.1 vector readings.

### 4.11. Statistical Analysis

We performed statistical analysis using Graph Pad Prism 9. The *p*-value summaries are as follows: *p* > 0.05 (ns); *p* ≤ 0.05 (*); *p* < 0.01 (**); *p* < 0.001 (***); *p* < 0.0001 (****). Statistical analysis for column graphs used multiple two-tailed *t*-tests using the Holm–Sidak method, with α = 0.05. qChIP values where the signal was more than twofold greater than the background anti-immunoglobulin G (anti-IgG) level were analyzed. Graphed data are presented as mean values, with bars representing the standard deviation (mean ± SD) of at least three technical replicates and at least two independent experiments.

## Data Availability

Newly generated data and reagents will be made available after appropriate MTA.

## Abbreviations

PI3K	phosphoinositide 3-kinase
IKZF1	Ikaros zinc finger 1
PIK3CD	phosphatidylinositol 4,5-bisphosphate 3-kinase catalytic subunit delta
PIKFYVE	phosphoinositide kinase, FYVE-type zinc finger containing
